# A Kalman Filter Implementation for Precision Improvement in Low-Cost GPS Positioning of Tractors

**DOI:** 10.3390/s131115307

**Published:** 2013-11-08

**Authors:** Jaime Gomez-Gil, Ruben Ruiz-Gonzalez, Sergio Alonso-Garcia, Francisco Javier Gomez-Gil

**Affiliations:** 1 Department of Signal Theory, Communications and Telematics Engineering, University of Valladolid, 47011 Valladolid, Spain; E-Mails: rruigon@ribera.tel.uva.es (R.R.-G.); salonsog@ribera.tel.uva.es (S.A.-G.); 2 Department of Electromechanical Engineering, University of Burgos, 09006 Burgos, Spain; E-Mail: fjggil@ubu.es

**Keywords:** Kalman filter, agricultural vehicle, Global Positioning System (GPS), vehicle guidance, sensor data fusion, autonomous navigation

## Abstract

Low-cost GPS receivers provide geodetic positioning information using the NMEA protocol, usually with eight digits for latitude and nine digits for longitude. When these geodetic coordinates are converted into Cartesian coordinates, the positions fit in a quantization grid of some decimeters in size, the dimensions of which vary depending on the point of the terrestrial surface. The aim of this study is to reduce the quantization errors of some low-cost GPS receivers by using a Kalman filter. Kinematic tractor model equations were employed to particularize the filter, which was tuned by applying Monte Carlo techniques to eighteen straight trajectories, to select the covariance matrices that produced the lowest Root Mean Square Error in these trajectories. Filter performance was tested by using straight tractor paths, which were either simulated or real trajectories acquired by a GPS receiver. The results show that the filter can reduce the quantization error in distance by around 43%. Moreover, it reduces the standard deviation of the heading by 75%. Data suggest that the proposed filter can satisfactorily preprocess the low-cost GPS receiver data when used in an assistance guidance GPS system for tractors. It could also be useful to smooth tractor GPS trajectories that are sharpened when the tractor moves over rough terrain.

## 1. Introduction

Global Positioning Systems (GPS) are nowadays used in many agricultural tasks [1–3]. GPS receivers with RTK differential corrections are frequently employed in agricultural equipment [4,5]. Nevertheless, tasks such as yield mapping [6] and assisted guidance in cereal fertilization do not always need centimeter precision. In consequence, some companies manufacture assisted guidance systems for tractors equipped with low-cost GPS receivers, such as *Agroguia*^®^ [7] and *Tractordrive*^®^ [8], for example, in Spain. Moreover, the universalization of mobile computing with smartphones and tablet devices, equipped with powerful processors and low-cost embedded GPS receivers, makes the use of these devices in agricultural tasks attractive. However, due to a quantization effect, most low-cost GPS receivers provide positions on a rectangular grid of some decimeters on each side. Because of this fact, low speed trajectories and parts of the trajectories with headings close to a coordinate axle suffer from significant speed, position, and heading errors when using low-cost GPS receivers.

The aim of this work is to smooth the tractor trajectories acquired by low-cost GPS receivers, by improving the precision of their position data by decreasing their quantization error. To do so, an implementation of the Kalman filter was applied to the trajectory data provided by a low-cost GPS receiver that was placed on a farm tractor.

The following points are introduced below for a better understanding of this implementation: (i) error considerations for GPS receivers; (ii) the quantization effects in low-cost GPS receivers; (iii) the kinematic model of a tractor; (iv) the Kalman filter; and (v) the Kalman filter tuning.

### 1.1. Error Considerations for GPS Receivers

**Figure 1.**
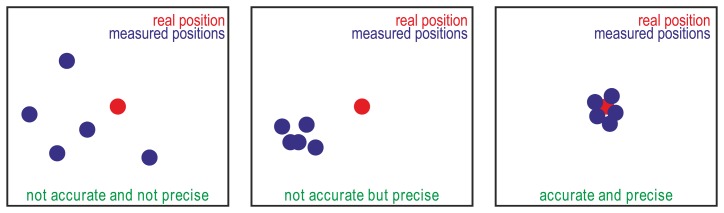
Graphic illustration of precision and accuracy concepts.

Two kinds of errors can be defined for GPS receivers and GPS guidance systems [9–11]: (i) precision, relative accuracy, reproducibility, repeatability, or pass-to-pass accuracy, which refer to the degree to which the measurements reported by a GPS receiver in a fixed placement provide close positions regardless of the real position; and (ii) accuracy or absolute accuracy, which refer to the degree of closeness of the measured positions to their real position. Figure 1 illustrates the difference between precision and accuracy.

In guidance system applications, where the time between each pass is relatively short and the trajectories are not saved from year to year, precision can be considered the most important variable. In this way, low-cost GPS receivers with 10 m accuracy but sub-meter precision will be alternatives in such agricultural task.

### 1.2. Quantization Effects in Low-Cost GPS Receivers

**Figure 2.**
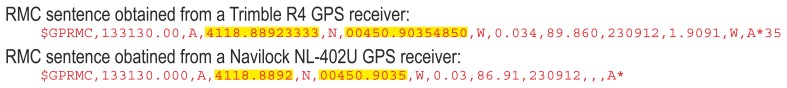
RMC Sentences acquired from two different GPS receivers. The yellow-highlighted numbers represent the latitude and longitude geodetic coordinates. It can be observed that the high end *Trimble R4* provides 12 digits for latitude and 13 for longitude while the low-cost *Navilock NL-402U* provides only eight digits for latitude and nine for longitude.

The most common chipsets that low-cost GPS receivers and embedded mobile computing devices integrate are the *Sirf* [12], the *U-blox* [13], and the *MTK* [14]. Receivers with either of these chipsets transmit positioning information by means of the National Marine American Association (NMEA) 0183 protocol, and provide latitude and longitude geodetic coordinates with usually only eight digits for latitude and nine for longitude (Figure 2).

**Figure 3.**
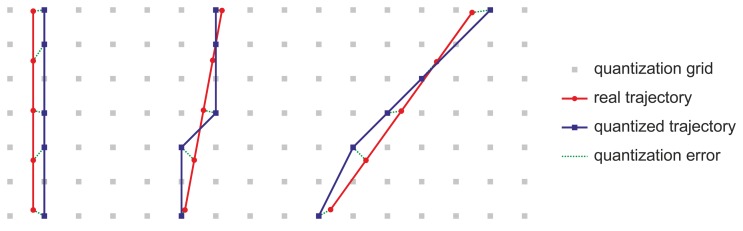
Illustration of the quantization effect on the positions supplied by a GPS receiver, showing that quantified trajectories register (i) position errors; and (ii) speed errors, as shown by the variable distances between the blue rectangles; and (iii) heading error, which are higher in trajectories nearby, but different from, the direction of any coordinate axis.

Geodetic coordinates are not appropriate for agricultural data processing and are usually converted to Cartesian coordinates. When positions in geodetic coordinates with a quantization of only 8 digits for latitude and 9 for longitude are converted to Cartesian coordinates such as Universal Transverse Mercator (UTM) [15–17] or East, North, Up (ENU) [18], they appear on a rectangular grid of some decimeters in size. Specifically, at the place where the real tests were conducted, with a latitude of 41.32° N and a longitude of 4.84° W, the quantization grid is 14 cm and 18 cm on the X and Y axes, respectively, in UTM coordinates. Position, speed, and heading errors appear in the trajectories on this grid, which provoke oscillations in the trajectory of the tractor. These oscillations are especially noticeable when they are acquired at low speeds and with headings close to the direction of a coordinate axis (Figure 3).

On the basis of their professional experience with *Agroguia*^®^ [7] and *Tractordrive*^®^ [8], the authors state that these oscillations negatively affect the use of low-cost GPS receivers in GPS assisted-guidance systems for tractors.

### 1.3. Kinematic Model of a Tractor

**Figure 4.**
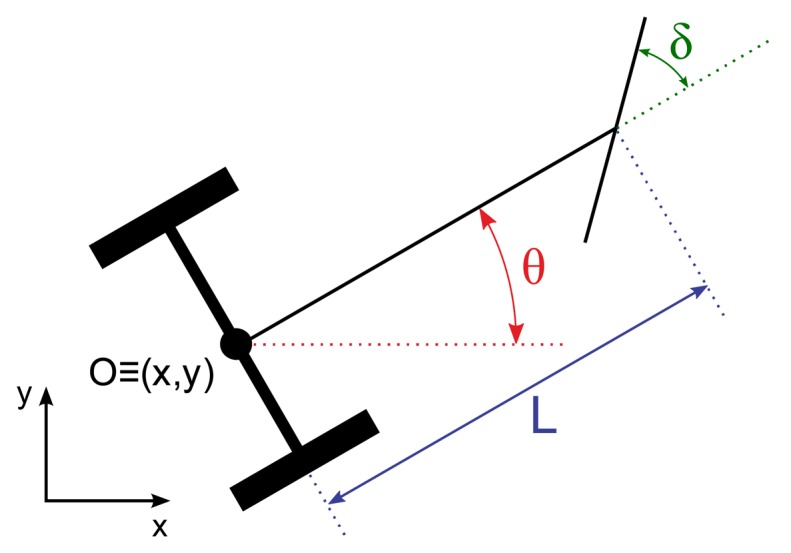
Tractor schematic and description of variables.

A classic tractor has two front wheels that steer as well as two rear wheels that are straight-driven. The behavior of this kind of tractor vehicle is typically modeled following the tricycle vehicle model [19]. In this model, the system inputs are the vehicle speed modulus, *u*, and the front-wheel steering angle, *δ*. The tractor behavior can be described with a vector state, ***q***, defined by the expression:

(1) $$q={\left[x,y,\theta ,u,\delta \right]}^{T}$$

and, with the equations of its kinematic model, assuming non-slip conditions on the wheels, given by:

(2) $$\begin{array}{c} \dot{x}=u\cdot \cos\theta \\ \dot{x}=u\cdot \sin\theta \\ \dot{\theta }=u/L\cdot \tan\delta \end{array}$$

where O ≡ (x,y) is the midpoint of the rear wheel axle, *x* and *y* represent the position in Cartesian coordinates of O, *θ* is the orientation of the vehicle with respect to the positive X-semiaxis, *δ* is the steering angle of the front wheels with reference to the vehicle's forward direction, and L is the length from O to the center of the front axle, *i.e.*, the distance between both axles. Figure 4 shows a schematic of the system and the variables.

### 1.4. The Kalman Filter

The Kalman filter is an efficient, recursive, mathematical algorithm that processes, at each step, inaccurate observation input data and generates a statistically optimal estimate of the subjacent real system state, by employing a prediction model and an observation model [20].

**Figure 5.**
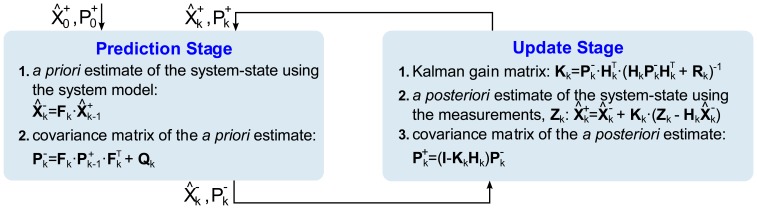
Stage diagram of the Kalman filtering loop.

The basic functioning of the filter is conceptualized into two stages. The first stage is called the prediction stage, as it produces an *a priori* system state estimate from the previous state, by using a system evolution prediction model. The second stage, known as the update stage, takes into account measurements in the system to produce an *a posteriori* state estimate, by correcting the previous *a priori* estimate. This two-stage process starts with an initial estimated state, ${\hat{x}}_{0}^{-}$, and is repeated in a loop recursively until filtering ends (Figure 5).

Figure 5 summarizes the steps in each stage of the Kalman filtering process and it presents the matrices that are involved and the steps followed to implement the Kalman filter [20,21]. ***F****_k_* is the state transition model matrix, which performs the prediction model. ***H****_k_* is the observation model matrix, which maps the state vector space into the measurements vector space. ${\hat{x}}_{k}^{-}$ is the *a priori* state estimate vector, resulting from the prediction stage. ${\hat{x}}_{k}^{+}$ is the *a posteriori* state estimate vector, derived from the measurements update stage. ***z****_k_* is the measurements vector obtained from the system sensors. ***K****_k_* is the optimal Kalman gain matrix, which weights the importance of the innovation that introduces the measurements vector *z_k_* in the update stage. $P_{k}^{-}$ is the *a priori* state covariance matrix, which provides the *a priori* estimation error covariance after the prediction stage. $P_{k}^{+}$ is the *a posteriori* state covariance matrix, containing the *a posteriori* estimation error covariance, given after the update stage. ***Q*** is the process noise covariance matrix of the prediction stage noise, which somehow ponders the weight of the process estimates. ***R*** is the observation noise covariance matrix of the update stage noise, which in a way ponders the degree of confidence in each one of the measurements. The relative weights become greater as the covariance matrix elements become smaller, meaning that the quantities involved are increasingly reliable.

### 1.5. The Tuning of the Kalman Filter

Kalman filter tuning consists of setting the relevant parameter values for the related noise covariance matrices ***Q***, ***R***, and $P_{0}^{+}$ [22]. Matrices ***Q***, ***R***, $P_{k}^{-}$, and $P_{k}^{+}$ reflect, respectively, the certainty or accuracy of the prediction model, the measurement model, the *a priori* prediction, and the *a posteriori* correction. The Kalman filter uses these matrices to weight the relevance and degree of confidence in predictions and measurements. The Kalman filter assumes that the involved noise characteristics have a zero-mean multivariate Gaussian distribution with covariance matrices ***Q*** and ***R*** for the process and measurements noises, respectively. Process noise is the random vector affecting the state ***x****_k_*, meanwhile measurement noise is the random vector affecting the measurements vector ***x****_k_*. Typically ***Q*** and ***R*** values must be estimated, in order to achieve statistically optimal filtering results. A covariance matrix contains the variance and cross-covariance information between each pair of elements of a random vector. In a general case, given a random vector, *Y*=[*Y*_1_*Y*_2_…*Y_n_*]*^T^*, its covariance matrix is expressed as:

(3) $$\Sigma =[\begin{array}{ccc} \Sigma_{1,1} & \cdots & \Sigma_{1,\text{n}}\\ \vdots & \ddots & \vdots \\ \Sigma_{\text{n},1} & \cdots & \Sigma_{\text{n},\text{n}} \end{array}],$$

where Σ_i,j_=cov(*Y_i_*, *Y_j_*)=*E* [(*Y_i_*− *η_i_*), (*Y_j_*− *η_i_*), *η_i_*= *E*[*Y_i_*], and *E* [·] denotes the expectation operator [23].

There are two main approaches to address the filter tuning: static and dynamic. Static tuning approaches only tune the filter just before its use. Static procedures are based on techniques such as the Autocovariance Least Squares (ALS) method [24], performance-convergence cost functions [25], and general numerical optimization methods [26]. On the other hand, dynamic or adaptive tuning approaches tune the filter while it is operating, thus providing a self-tuning capability. Dynamic tuning methods are based on techniques such as Fuzzy Logic (FL) [27], Artificial Neural Networks (ANN) [28], Reinforcement Learning (RL) [29], the Dynamic Error System Analysis (DESA) method [30], and Genetic Algorithms (GA) [31,32]. Adaptive methods tend to yield a more robust behavior in cases such as those where noise characteristics change in time.

## 2. Method

This section comprises the work carried out in this survey. Section 2.1 outlines the particularization of the Kalman filter along with the prediction model that is employed. Section 2.2 deals with the method employed to tune the filter, in order to achieve a suitable performance with artificial data. Section 2.3 explains the experimental system employed in real field tests, to check the behavior of the proposed system.

### 2.1. Kalman Filter Particularization in Tractor Guidance

The system presented in this study uses a particularization of the Kalman filter applied to GPS receiver data, in order to achieve path smoothing and partial restoration of the lost resolution in positioning data. The prediction model of this system is based on the tricycle kinematic model, seen in Equation (2), assuming that the vehicle speed and heading angle will change slowly. Additionally, the system also makes use of the measurements provided by a GPS receiver to update the predictions previously made.

**Figure 6.**
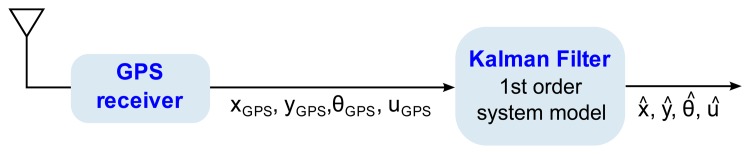
Black box diagram of the system implementation.

The system takes the array of GPS measurements **z***_k_*(*x_GPS_*, *Y_GPS_*, *θ_GPS_*,*u_GPS_*)*^T^*, as input variables, where *x_GPS_* is the position in the X-axis, *y_GPS_* is the position in the Y-axis, *θ_GPS_* is the measured heading angle formed by the tractor heading and the positive X-semiaxis, and *u_GPS_* is the speed modulus of the tractor. Given these inputs, ***z****_k_*, to the system, a related system state-vector is defined as ***x****_k_* = (*x_k_*,*y_k_*, *θ_k_*,*u_k_*)*^T^*, which will be estimated by the Kalman filter. Figure 6 shows a black box diagram of this particular Kalman filter implementation.

Based on the system state, ***x****_k_*, along with Equation (2), a prediction model is applied, which supposes that vehicle heading angle (*θ*) and speed (*u*) will change slowly. The *a priori* state estimate is obtained from the previous *a posteriori* state estimate as:

(4) $$x_{k}^{-}=x_{k-1}^{+}+u_{k-1}^{+}\cdot \Delta T\cdot \cos\theta_{k-1}^{+}$$

(5) $$y_{k}^{-}=y_{k-1}^{+}+u_{k-1}^{+}\cdot \Delta T\cdot \sin\theta_{k-1}^{+}$$

(6) $$\theta_{k}^{-}=\theta_{k-1}^{+}$$

(7) $$u_{k}^{-}=u_{k-1}^{+}$$

where the subscript *k* denotes the discrete epoch at which the calculations are computed, *ΔT* denotes the time lapse between the reception of two successive position data vectors, and the *a priori* and *a posteriori* state estimates are denoted, respectively, by a minus or a plus sign in superscript. Each of these discrete times matches the sampling time when the GPS receiver emits a new positioning sentence.

Labeling the a priori state estimate vector as ${\hat{x}}_{k}^{-}={\left(x_{k}^{-},y_{k}^{-},\theta_{k}^{-},u_{k}^{-}\right)}^{T}$ and the a posteriori state estimate vector as ${\hat{x}}_{k}^{+}={\left(x_{k}^{+},y_{k}^{+},\theta_{k}^{+},u_{k}^{+}\right)}^{T}$, Equations (4)–(7) are rewritten into the matrix form of the Kalman filter as:

(8) $${\hat{x}}_{k}^{-}=F_{k}{\hat{x}}_{k-1}^{+}$$

where, ***F****_k_* denotes the prediction matrix:

(9) $$F_{k}=[\begin{array}{cccc} 1 & 0 & 0 & \Delta T\cdot \cos\theta_{k-1}^{+}\\ 0 & 1 & 0 & \Delta T\cdot \sin\theta_{k-1}^{+}\\ 0 & 0 & 1 & 0\\ 0 & 0 & 0 & 1 \end{array}]$$

It may be seen from Equation (9) that the prediction matrix has to be updated at every step, since the variable $\theta_{k}^{+}$ can change within each iteration, and the prediction matrix ***F****_k_* depends on it in a non-linear way, which is not accounted for in the ***F****_k_* matrix.

It is necessary to define the observation model matrix, ***H****_k_*, in Equation (10), for the complete characterization of the proposed Kalman filter instance:

(10) $${\hat{x}}_{k}^{+}={\hat{x}}_{k}^{-}+K_{k}(z_{k}-H_{k}{\hat{x}}_{k}^{-})$$

As the system state vector and measured magnitudes perfectly match, the particular observation model matrix ***H****_k_* is chosen as the identity matrix in Equation (11):

(11) $$H_{k}=I_{4}=\left[\begin{array}{cccc} 1 & 0 & 0 & 0\\ 0 & 1 & 0 & 0\\ 0 & 0 & 1 & 0\\ 0 & 0 & 0 & 1 \end{array}\right],$$

where ***I***_4_ denotes the identity matrix of size 4 × 4.

### 2.2. Procedures

The Kalman filter was particularized for tractor guidance as detailed in Section 2.1, and the ***F*** and ***H*** system model matrices were obtained. Assuming statistical independence between all state variables, and assuming that the covariance matrices are time-invariant, the ***Q*** and ***R*** covariance matrices can be represented as Equation (12):

(12) $$Q=\left[\begin{array}{cccc} q_{1,1} & 0 & 0 & 0\\ 0 & q_{2,2} & 0 & 0\\ 0 & 0 & q_{3,3} & 0\\ 0 & 0 & 0 & q_{4,4} \end{array}\right]\;R=\left[\begin{array}{cccc} r_{1,1} & 0 & 0 & 0\\ 0 & r_{2,2} & 0 & 0\\ 0 & 0 & r_{3,3} & 0\\ 0 & 0 & 0 & r_{4,4} \end{array}\right]$$

**Figure 7.**
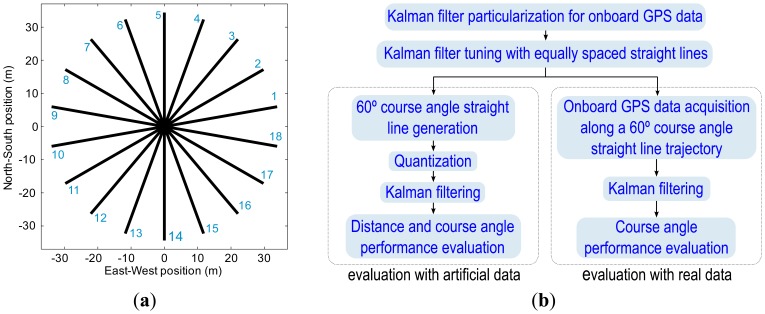
(**a**) Paths used in the tuning process and distance performance evaluation; (**b**) Flow charts of Kalman filter evaluation with artificial data and with onboard GPS real data.

**Figure 8.**
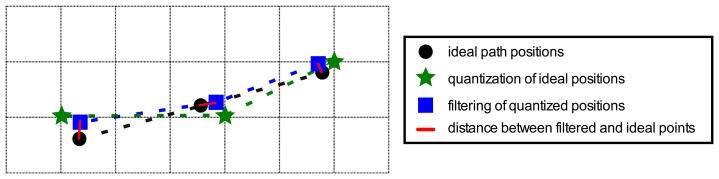
Illustration of the quantization and Kalman filter process conducted over an ideal sample trajectory. The RMSE is defined, according Equation (13), as the square root of the average of all the distances squared with respect to the real reference path position.

The Kalman filter was tuned using Monte Carlo Sampling techniques [33], by repeating the following two steps two million times. Initially, the elements of covariance matrices ***Q*** and ***R*** were randomly chosen between 0 and 6. Secondly, the particularized Kalman filter, using these covariance matrices, was applied over the 18 straight lines in Figure 7a, and then the Root Mean Square Error (RMSE) was computed, following Equation (13), over all straight lines, in which ***N*** is the number of samples, ***x̂**_i_* the *i-th* estimate of the X axis position, ***ŷ**_i_* the *i-th* estimate of the Y axis position, and ***x*** and ***y*** are the ideal position reference coordinates. The couple of matrices with the lowest Root Mean Square Error were taken as covariance matrices ***Q*** and ***R***. Figure 8 and Equation (13) detail the Root Mean Square Error (RMSE) computing procedure.

(13) $$\text{RMSE}=\sqrt{\frac{1}{N}\sum_{i=1}^{N}{\left({\hat{x}}_{i}-x_{i}\right)}^{2}+{\left({\hat{y}}_{i}-y_{i}\right)}^{2}}=\sqrt{\frac{1}{N}\sum_{i=1}^{N}{{\text{distance}}_{i}}^{2}}$$

The proposed Kalman filter performance was evaluated with artificial data, through the following steps (Figure 7b): (i) a set of 18 ideal straight trajectories (Figure 7a) were sampled at a 5 Hz update rate, following vehicle kinematics constraints, at a constant speed of 5 km/h, which is a typical speed for agricultural tasks; (ii) trajectories were quantized to a 14 × 18 cm grid, because in Valladolid, Spain, when low-cost GPS receivers that provide eight digits for latitude and nine for longitude in NMEA are employed, positions appears over this grid; (iii) the proposed Kalman filter model was applied to the quantized paths; and (iv) the performance improvements were evaluated in terms of distance with respect the ideal path using all the paths shown in Figure 7a, and in terms of course angle distribution by using a 60° heading angle straight path.

Finally, the proposed Kalman filter performance was evaluated with real GPS data by following the next steps (Figure 7b): (i) GPS receiver data were acquired at a 5 Hz update rate from a GPS placed on a tractor that traveled along straight path with a 60° heading angle, and the GPS positions were converted to UTM coordinates; (ii) the proposed Kalman filter was applied to the acquired data; and (iii) the performance achievements in course angle distribution were evaluated. All the required simulations and data processing were carried out in MATLAB^®^ programming environment.

### 2.3. Experimental System

**Figure 9.**
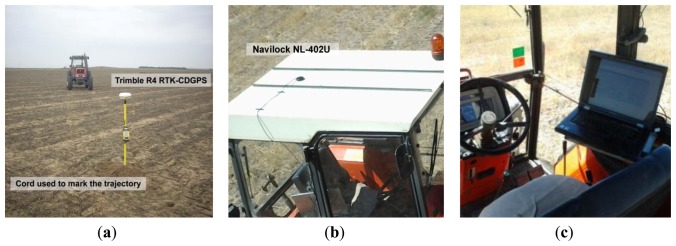
(**a**) One of the trajectories of the tests with one stake and the cord; (**b**) Low-cost GPS receiver placed over the tractor cab; (**c**) Laptop inside the tractor cab.

The materials employed in the experimental tests of this article were: a low-cost GPS receiver, a precise GPS receiver, a laptop computer, and an agricultural tractor. The low-cost GPS receiver was a *Navilock NL-402U* with an *U-blox LEA-5H* chipset (Figure 9b), and it was employed to acquire the GPS trajectories to be processed in this article at a 5 Hz rate. A precise *Trimble R4* GPS receiver, configured to use RTK corrections, was employed to position the stakes that were used to mark the paths in the plot (Figure 9a). The laptop was a *Lenovo N3000* (Figure 9c) and it was used to acquire and store the trajectories from the low-cost GPS receiver. The agricultural tractor was a *Kubota M6950DT* (Figure 9a), and it was employed to perform the trajectories with the low-cost GPS receiver over its cab (Figure 9b).

Each one of the straight trajectories of the experimental tests was marked with three stakes and were joined by a cord. The stakes were driven into the center of the trajectory and at each extreme. Figure 9a shows one of the trajectories and one stake.

GPS receiver data were read and processed with an application running on the laptop. The application, developed using *Labwindows* CVI, read the NMEA sentences from the GPS receiver and transformed the geodetic data of the NMEA sentences to UTM Cartesian coordinates for analysis.

## 3. Results

The tuning results, following the method presented in Section 2.2, were obtained using: (i) the artificially generated paths (Figure 7a) with a constant speed of 5 km/h; (ii) a 14 × 18 cm resolution quantization grid; (iii) an update rate of 5 Hz; (iv) the Kalman filtering model proposed in Section 2.1; and (*v*) the Root Mean Square Error minimizing criterion, defined in Section 2.2. The process noise covariance matrix, the measurements noise covariance matrix, and the *a posteriori* state covariance matrix were as follows:

(14) $$\begin{array}{c} Q=\left[\begin{array}{cccc} 0.23 & 0 & 0 & 0\\ 0 & 0.26 & 0 & 0\\ 0 & 0 & 0.01 & 0\\ 0 & 0 & 0 & 1.05 \end{array}\right],\\ R=\left[\begin{array}{cccc} 1.51 & 0 & 0 & 0\\ 0 & 5.58 & 0 & 0\\ 0 & 0 & 1.95 & 0\\ 0 & 0 & 0 & 1.68 \end{array}\right],\\ P_{0}^{+}=[\begin{array}{cccc} 3.7 & 0 & 0 & 0\\ 0 & 6.4 & 0 & 0\\ 0 & 0 & 3.7 & 0\\ 0 & 0 & 0 & 6.7 \end{array}]. \end{array}$$

These matrices, Equation (14), resulting from Kalman filter tuning, were used for both the simulations with artificial data and the real experimental data obtained from the onboard GPS receiver.

**Figure 10.**
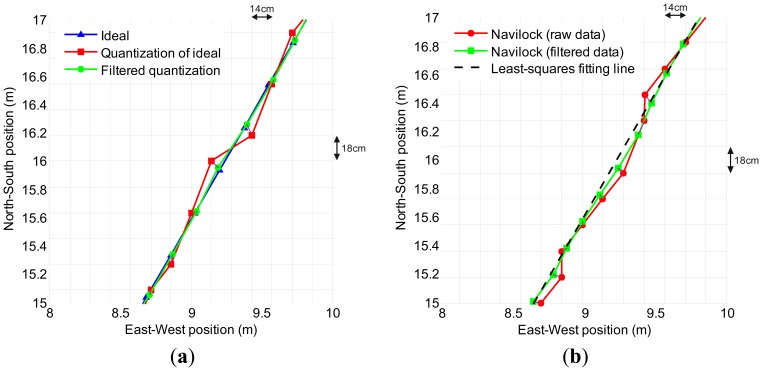
Results of positioning improvement at a 5 Hz update rate, constant speed of 5 km/h (3.1 mph) and 60° heading angle along a straight path (**a**) in a simulation with artificial data; and (**b**) in tests, processing data from a real onboard *Navilock NL-402U* GPS receiver. The purple line joins two corresponding points, before and after the filtering, to qualitatively show the negligible magnitude of the delay.

Simulations and real tests were executed to evaluate the Kalman filter performance. As Figure 10 visually illustrates, significant trajectory smoothing and resolution restoration achievements were accomplished in both situations, and no delays are noticed in either simulations or real tests. The achievements are shown along a selected path with a 60° heading angle, for both an artificially generated straight line path and onboard GPS receiver real data.

**Figure 11.**
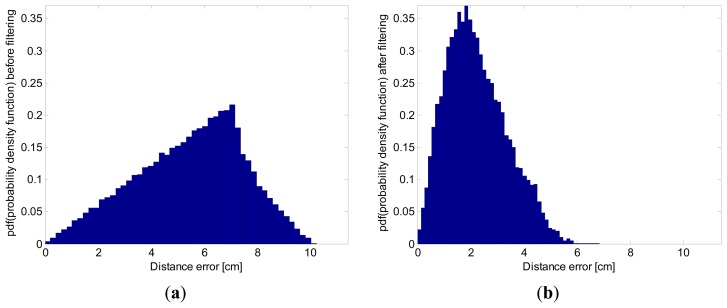
Histogram of distance errors, with 5 Hz update rate, using the simulations along the 18 straight lines shown in Figure 7a. (**a**) before applying the Kalman filter; (**b**) after applying the Kalman filter. The histogram has been normalized so that it has a unitary area, representing an approximation to the probability density function (*pdf*) of the distance errors statistical random variable.

**Figure 12.**
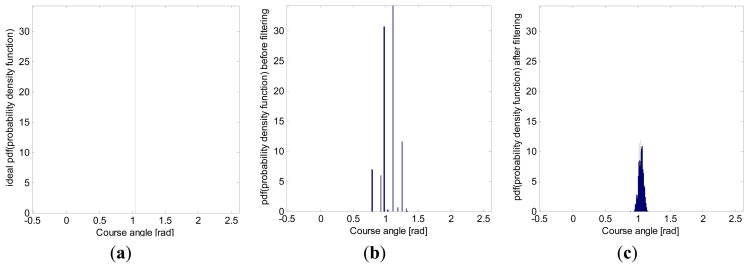
Heading angle (*θ*) histogram, (**a**) of the real ideal path; (**b**) of the grid-quantized path; and (**c**) of the recovered path after applying the proposed Kalman filter. Simulated data were obtained for a straight path with a 60° heading angle and at varying speeds of between 5 and 10 km/h. The histogram has been normalized to have a unitary area, representing an approximation to the probability density function (*pdf*) of the heading angle statistical random variable.

The properties and improvements of this proposed method are shown with the distance errors histogram (Figure 11), the RMSE and the 95*th*-percentile of the distance errors (Table 1), and the heading angle histogram (Figure 12).

Figure 11 presents the distance error histogram with regard to real reference positions, before and after filtering. These histograms were generated with the data from all the paths in Figure 7a. It is observed that, before applying the proposed Kalman filter, there are distance errors of up to 10 cm whereas, after applying the Kalman filter, the distance errors go no higher than 6 cm (Figure 11).

The RMSE and the 95*th*-percentile were computed, on the basis of the statistical distribution of the distance errors, as shown in Figure 11. These measurements can be employed to quantify the error improvement achieved by the proposed filtering. Table 1 refers to the RMSE of the quantization error, which was reduced by 42.98%.

Another illustrative graph, showing the behavior of the filtering along straight lines, is the heading angle histogram. As Figure 12 shows, the proposed Kalman filter meaningfully reduced the spread from the real heading angle.

The standard deviation and the 95*th*-percentile range, computed from the heading angle histogram in Figure 12, are shown in Table 2, in which the standard deviation was reduced by 73.62% in the simulations with artificial data.

**Figure 13.**
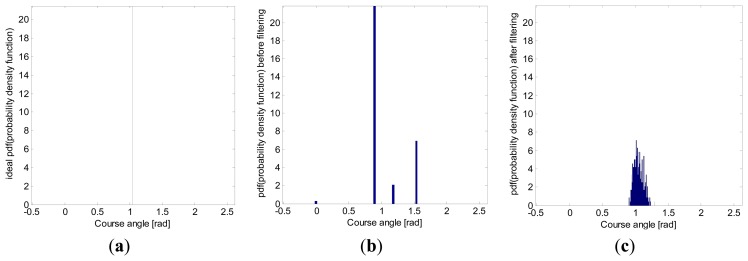
Heading angle (*θ*) histogram (**a**) of the estimated reference path; (**b**) of the raw GPS data; and (**c**) of the filtered data using the proposed Kalman filter. Data were obtained from a real onboard *Navilock NL-402U* GPS receiver, along a straight path with a 60° heading angle and at a constant speed of 5 km/h. The histogram has been normalized to have a unitary area, representing an approximation to the probability density function (*pdf*) of the heading angle statistical random variable.

Another heading angle histogram was also computed, this time with experimental data acquired from a low-cost *Navilock NL-402U* GPS receiver (Figure 13). As with simulations, the filtering also avoided great changes of heading angle, thus achieving a smoother path.

The standard deviation and the 95*th*-percentile range, computed from the heading angle histogram in Figure 13, are shown in Table 3. As this table shows, the standard deviation was also reduced by around 75.04% in the real field tests.

## 4. Discussion

The main finding of the present study is that implementation of the Kalman filter can reduce the quantization errors in the positioning of tractors equipped with some low-cost GPS receivers by 43%. Moreover, it reduces by 75% the standard deviation of the heading angle.

Several studies have shown that GPS accuracy can range from 1–2 cm to 100 m [34–36] depending on the kind of GPS receiver and of the type of corrections employed. For low-cost GPS receivers using Wide Area Augmentation System (WAAS) or European Geostationary Navigation Overlay Service (EGNOS) corrections, position accuracy of 95% time can be less than 3 m [37–39]. In contrast, relative accuracy or pass-to-pass accuracy [10] is the really important variable for multiple agricultural applications. In experimental tests, Alonso-Garcia *et al.* [40] found that this relative accuracy can be reduced to approximately 1 m over short time periods of about 15 min, when using low-cost GPS receivers. This relative accuracy could be enough for agricultural applications with wide working widths, such as fertilizing applications. In fact, some companies, such as *Agroguia*^®^ [7] and *Tractordrive*^®^ [12] in Spain, sell tractor GPS guidance assistance systems equipped with low-cost GPS receivers.

In a previous study, this research team has proposed a way of improving the precision of GPS tractor positioning [41]. Moreover, the authors have acquired expertise in their work on tractor GPS guidance systems with two Spanish companies: *Agroguia*^®^ [7] and *Tractordrive*^®^ [12]. In their experience, the GPS receivers with the best price-precision ratios for agricultural tasks, on the market from 2008–2013, were low-cost GPS receivers equipped with *Sirf IV* and *U-blox 4* chipsets. Nevertheless, GPS receivers equipped with these chipsets offer geodetic latitude and longitude with only 8 and 9 digits, respectively. When these coordinates are converted to Cartesian coordinates by the GPS guidance system, the positions fit in a quantization grid, the dimensions of which vary in accordance with the point on the terrestrial surface. In Valladolid, Spain, the grid is about 14 and 18 cm, on the X and the Y axes, respectively. Tractor guidance systems that employ positions within this grid suffer from oscillations in the representation of the tractor trajectory. Besides, the GPS data with heading oscillation complicate the steering of the tractor. The proposed Kalman filter is especially useful for tractor guidance assistance systems equipped with GPS receivers that have *Sirf IV* and *U-blox 4* chipsets. It smooths the quantized sharp trajectory and provides more accurate heading information, closer to the real data, thereby facilitating the steering of the tractor.

Tractors usually move over rough surfaces, and then, although a tractor goes along a straight trajectory, the GPS receiver will acquire a sharp trajectory. This is due to the lateral vibrations experienced by the GPS receiver, which is placed over the tractor cab, two or three meters above-ground. At this position, the vertical displacement of the tractor wheels is converted into lateral vibrations. As a numerical example, a typical 10 cm vertical displacement of one rear wheel of the tractor can lead to a lateral displacement of the GPS receiver of up to 30 cm. Our implementation of the Kalman filter will be useful in these situations, because it will smooth the sharp trajectory due to vibrations and will provide more accurate heading information, close to the real data. Similar studies to remove noise from GPS data have been presented in the literature [42]. The main difference of our article and the one proposed by Han *et al.* [42] is the Kalman filter tuning mode; Han *et al.* tuned it by trial and error whereas we applied *Monte Carlo* techniques to eighteen trajectories to obtain the covariance matrices that produce the lowest Root Mean Square Error.

Overall, our data suggest that the proposed filter is adequate for data preprocessing of some low-cost GPS receivers, when used in GPS assisted-guidance systems for tractors. It also could be useful to smooth the GPS trajectories that are sharpened due to the tractor moving over rough terrain.

One limitation of this study is that the proposed Kalman filter reduces the quantization error in GPS receivers that provide geodetic latitude and longitude with eight and nine digits, but, in GPS receivers that provide more digits, the reduction in the quantization error does not exist or is negligible. Microelectronics technology progresses quickly. Hopefully, in a few years all GPS receivers in the market, high end and low cost, will be equipped with chipsets that provide positioning data with enough digits, so that the quantization effects are negligible. Nevertheless, today, our proposed Kalman filter is at present useful for processing the data of some low-cost GPS receivers. Moreover, in the future the filter will be useful for the preprocessing of GPS trajectories on tractors moving over rough surfaces. A second drawback of our proposed Kalman filter is that, besides smoothing errors, as a side effect real deviations are also smoothed. This effect could be negative in some systems, as for example the used in GPS assisted guidance of tractors.

Further studies could be conducted. A more detailed quantitative analysis about inherent delays of the proposed system, both along both straight and curve paths, could be addressed. Besides, because most low-cost GPS receivers provide positioning information at 1 Hz rate, simple modifications to the Kalman filter proposed in this paper could be employed to increase the positioning rate. Modifications could also be used to fuse the data from low-cost GPS receivers with local positioning systems as gyroscopes and compasses employing, for example, *Arduino*^®^ or *Raspberry Pi*^®^ boards. Since nowadays most modern smartphones also include gyroscopes and compasses, it will be possible to deploy this system using a smartphone that fuses the data from its own sensors.

## 5. Conclusions

In summary, certain low-cost GPS receivers, such as those equipped with *Sirf IV* and *U-blox 4* chipsets, offer positioning information by using NMEA with eight digits for geodetic latitude and nine for longitude. When these geodetic coordinates are converted into Cartesian coordinates, the positions fit in a quantization grid. The dimensions of the quantization grid vary for each point on the terrestrial surface and usually range some decimeters in size. The Kalman filter implementation in this study, applied to data from these low-cost GPS receivers, has reduced the quantization errors by 43% and the standard deviation of the heading by 75%, without introducing positioning delays. On the basis of our data, we consider that use of the filter improves the precision of low-cost GPS receivers in some agricultural tasks, such as GPS assisted-guidance of tractors. It also could be useful to smooth tractor GPS trajectories that are sharpened when the tractor moves over rough terrain.

## Figures and Tables

**Table 1.** Statistical parameters of the distribution of errors, before and after applying the proposed Kalman filter, along the 18 simulated straight paths (Figure 7a).

	**Before Filtering**	**After Filtering**
Distances Root-Mean Square Error (RMSE) (cm)	6.56	3.74
Distances 95*th*-percentile (cm)	8.48	4.31

**Table 2.** Statistical parameters of the heading angle distributions, before and after applying the proposed Kalman filter, along a straight path with a 60° heading angle, in simulations with artificial data.

	**Before Filtering**	**After Filtering**
Standard deviation (°)	6.9362	1.8301
95*th*-percentile range (centered on real heading angle) (°)	29.0661	7.2651

**Table 3.** Statistical parameters of the heading angle distribution, before and after applying the proposed Kalman filter, along a straight path with a 60° heading angle, in real field tests.

	**Before Filtering**	**After Filtering**
Standard deviation (°)	16.5594	4.1326
95*th*-percentile range (centered on reference heading angle) (°)	55.1185	14.7193
